# Nasal continuous positive airway pressure with head cap fixation as a contributing factor to extensive scalp necrosis in a preterm neonate with early-onset sepsis and scalp hematoma

**DOI:** 10.1186/s12887-019-1721-2

**Published:** 2019-10-25

**Authors:** P. Zachhau, A. E. Gravergaard, H. T. Christesen

**Affiliations:** 1 Dept. Obstetrics and Gynecology, Odense University Hospital, Odense, Denmark; 2 Dept. of Plastic Surgery, Odense University Hospital, Odense, Denmark; 3 Hans Christian Andersen Children’s Hospital, Odense University Hospital, Odense, Denmark

**Keywords:** Nasal CPAP, Early onset sepsis, Scalp necrosis, Preterm

## Abstract

**Background:**

Nasal continuous positive airway pressure (CPAP) is widely used in the treatment and prevention of respiratory distress in preterm neonates, with only few severe adverse skin effects reported.

**Case presentation:**

A preterm neonate was born at 34 + 1 weeks of gestation, birth weight 1860 g, and presented with early-onset sepsis (EOS) and scalp hematoma. He developed respiratory distress day 2 after birth. Antibiotics, nasal CPAP and other supportive treatment were initiated. A scalp hematoma in the occipital region was complicated by nasal CPAP cap pressure leading to an extensive scalp necrosis equaling 6% of the total body surface. Debridement and skin grafting were performed day 11, and 51, respectively. The boy survived with good healing of the skin graft.

**Conclusion:**

The nasal CPAP head cap contributed to the development of severe, but potentially preventable, scalp necrosis in a preterm with birth-related scalp skin injury and EOS.

## Background

Nasal continuous positive airway pressure (CPAP) is a well-established noninvasive form of respiratory care, widely used as prevention or treatment of respiratory distress in preterm infants [1–3]. Nasal CPAP supports the spontaneous breathing by improving oxygenation, reducing airway resistance, improving the synchrony of the thoracoabdominal movements, and enhancing the inflation reflex [1, 2]. The nasal CPAP is usually well tolerated, but adverse effects such as gastric distension, pneumothorax, nasal complications, and skin trauma occur [1, 2]. We present the first report of use of nasal CPAP with head cap as a contributing factor for the development of severe scalp necrosis in a preterm neonate with early-onset sepsis (EOS) and scalp hematoma.

## Case presentation

The boy was born at 34 + 1 weeks of gestation, birth weight 1860 g (− 2 SD), to a 36-year-old gravida 4, para 1. In late pregnancy, the mother was diagnosed with group B streptococcus and treated with oral antibiotics. Premature rupture of membrane led to hospitalization for close monitoring using cardiotocography and fetal scalp electrode. Penicillin G was given twice intravenously and oxytocin was administrated continuously for stimulation of contraction. Vaginal examination showed a straight occipito-posterior presentation. Because of lack of progression in labor, vacuum-assisted labor was tried twice followed by acute cesarean. Apgar score was 1/9 and 5/10, umbilical cord pH 7.24, and Base Excess − 4.5 mmol/L.

After birth the infant was transferred to the in-house neonatal department. On day 2, staring, hypertonia, respiratory distress, apnea, and increased gastric residuals were observed, along with a gradually increasing, firm swelling in the occipital region of the scalp. No convulsions were observed. Treatment with bi-nasal flow CPAP (appropriate size Argyle® short nasal cannula fixed with a cotton head cap, Benviniste ventile, heated and humidified air, 5–6 cm H_2_O pressure), caffeine, intravenous ampicillin, and gentamycin was commenced. Cefotaxime was added to the treatment because of E-coli in both blood and cerebrospinal fluid. The respiratory distress along with blood gas values gradually improved. The results of repeated ultrasound scans of cerebrum and abdomen along with echocardiography were normal.

**Fig. 1:**
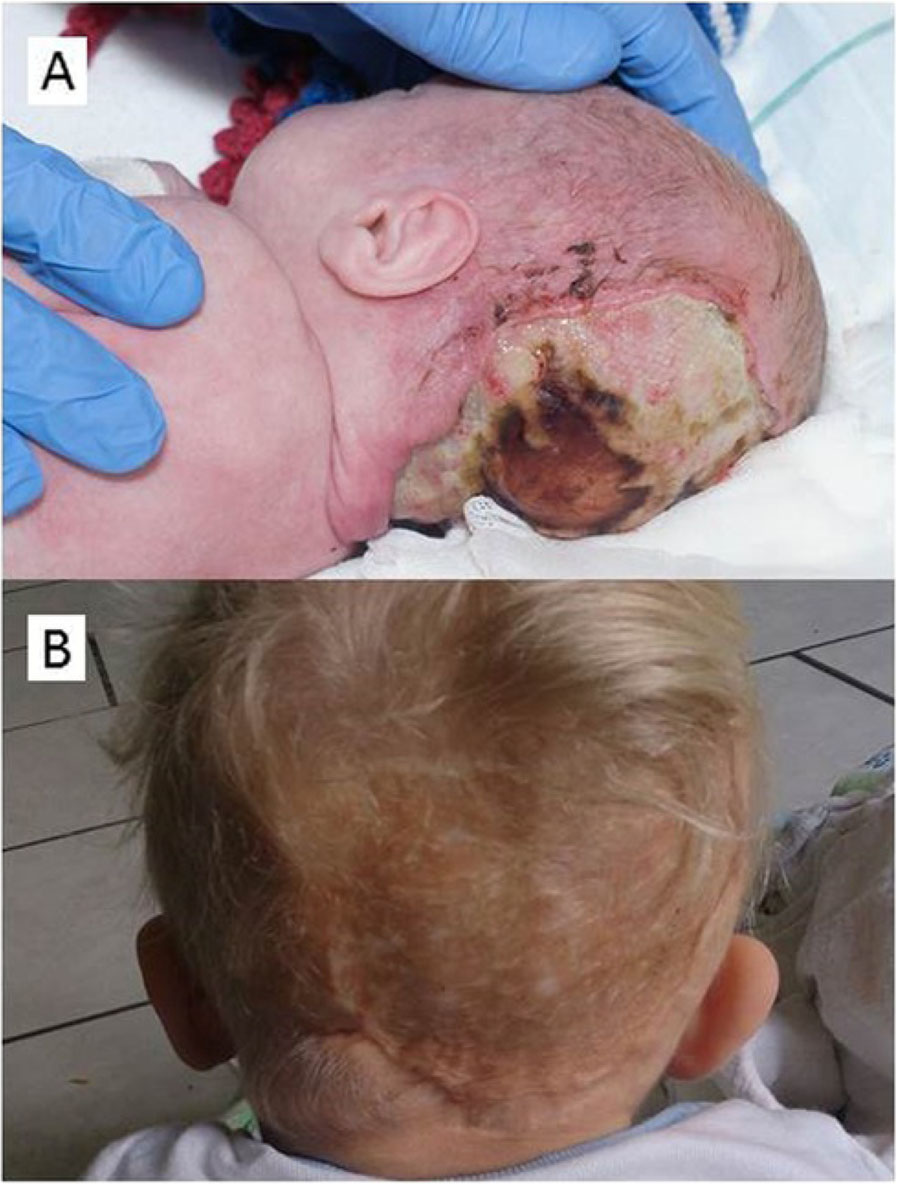
Extensive scalp necrosis before (**a**), and after surgery (**b**)

No signs of skin infection were initially registered. On day 3, skin darkening in the occipital region of the scalp was observed. From day 4 to day 6, the skin in the line of the CPAP cap on the right side of occipital area became increasingly dark and hard, and a small ulceration in this area was observed. During day 8 a spontaneous rupture of a part of the dark skin area followed by secretion of pus was observed. The CPAP was discontinued, the cap removed, and a plastic surgical assessment was made. During day 9 and day 10, a defect and increasing maceration of the skin in the occipital area gradually developed (Fig. 1A). In this period leucocytes increased to 37.1 E10^9^/L, while C-reactive protein had a biphasic course. On day 11, a reassessment of the skin was made, and debridement of the skin lesion in the occipital area equaling 6% of the total body surface was carried out during general anesthesia. The necrotic skin along with surrounding healthy tissue was removed, saving the periosteum for possibility of later skin grafting. Antibiotic treatment was reduced to cefotaxime, and the infant’s clinical condition as well as infection parameters improved. On day 51, skin grafting was successfully performed using skin from the back. Good healing of the skin graft was observed at surgical follow-up, apart from a small degree of tightening of the graft and occasional wounds (Fig. 1B).

## Discussion

Our patient developed an extensive scalp necrosis as a result of a combination of CPAP treatment cap pressure, EOS and scalp hematoma.

The skin matures around week 33 of gestation, but is at this time thin, vulnerable, and has increased transdermal water loss [4, 5]. Although the skin in preterm infants matures within few weeks after birth, special attention to skin care and skin lesions is needed [4, 5]. Significant risk factors for skin injuries in neonates include low birthweight, low gestational age, skin texture, limited number of position changes, and nasal CPAP [4, 5]. In general, skin lesions as a result of nasal CPAP treatment are mild and a rather common complication affecting 20 to 60% of the patients, whereas severe skin lesions with necrosis are rare [6, 7]. Severe skin lesions primarily affect the nose, while few lesions in the occipital region and in the forehead have been reported [4–7]. In Denmark, nasal CPAP has been widely used for more than 30 years [8] with no reported major skin injuries. Local-made, hospital washed, appropriate size 100% cotton head caps with bands tightened below the chin have been used routinely and exchanged once daily with inspection for skin injuries as for our patient. The neonatal database from our ward counted 845 preterm infants (age < 32 weeks or birth weight < 1500 g) treated with CPAP from 2006 to 2015. Of those, 277 had sepsis and only the present patient had severe skin complications.

Scalp hematomas occur in up to 4% of vacuum or forceps deliveries, but only in rare cases scalp hematomas become infected, most commonly by E-coli, Staphylococcus and Streptococcus species [9, 10]. Infected scalp hematomas may occur more often after needle aspiration, fetal monitoring, and instrumental delivery, as in our patient. Infected scalp hematoma should be suspected with increasing scalp hematoma, erythema, fluctuation, and relapse of a systemic infection. Clear signs of abscess in the scalp should be treated with incision, aspiration, antibiotics covering the most common microbes present in scalp hematomas, and often followed by surgical debridement [9, 10]. On day 2 our patient developed a scalp hematoma with no early signs of infection. The use of nasal CPAP with head cap from day 2 was judged to contribute to the development of the skin infection and necrosis by skin pressure, as well as delay in diagnosis, as the skull was inspected during cap changes only once daily.

Guidelines for the prevention and early detection of skin trauma of the scalp, ears and nostrils are essential for the use of nasal CPAP. Some studies suggest that general skin assessment should be carried out no less than every 4 h as a part of preventive care [4, 5]. In this process, the Braden Q score is suggested as a useful instrument in evaluating the risk of pressure ulcers [5]. Preventive care with gel pillows, foam mattresses, skin barrier products, and correction of position should be taken in consideration [4, 5]. Regarding nasal CPAP, the importance of adequate size of the CPAP equipment is stressed, as well as the proper placement of the equipment on the infant [4–6]. Other possibilities to potentially prevent nasal CPAP-related skin injuries include other fixation devices such as comfort wrap headgear to allow easier inspection, single nasal prong and nasal high flow support. Skin injuries should be managed with repositioning of the infant, barrier protection, respiratory care, antibiotics, and plastic surgical assistance [4, 5]. Our report highlights the importance of close observation and skin care in preterm infants with nasal CPAP, especially in case of EOS and scalp injury at birth.

## Conclusion

In preterm neonates with birth-related scalp skin injury and EOS, nasal CPAP with head cap may contribute to the development of extensive scalp necrosis. This severe complication is potentially preventable by regular skin inspection and skin care, as well as appropriate positioning of the CPAP cap. If signs of major skin injury occur, expert plastic surgical assistance should be acquired.

## Data Availability

All relevant data are included within the manuscript.

## Abbreviatons

CPAP: continuous positive airway pressure.
EOS: Early-onset sepsis.

## References

[1] Diblasi RM (2009). Nasal continuous positive airway pressure (CPAP) for the respiratory Care of the Newborn infant. Respir Care.

[2] Chowdhury O, Wedderburn CJ, Duffy D, Greenough A (2012). CPAP Review. Eur J Pediatr.

[3] Sweet DG, Carnielli V, Greisen G (2019). European consensus guidelines on the Management of Respiratory Distress Syndrome - 2019 update. Neonatology..

[4] Fujii K, Sugama J, Okuwa M, Sanada H, Mizokami Y (2010). Incidence and risk factors of pressure ulcers in seven neonatal intensive care units in Japan: a multisite prospective cohort study. Int Wound J.

[5] Ottinger D, Hicks J, Wilson S, Sperber K, Power K (2016). The pressure is on!, Neonatal skin and Nasal Continuous positive Airway Pressure. Adv Neonatal Care.

[6] Hogelin M, Fardin SR, Frieden IJ, Wargon O (2012). Forehead pressure necrosis in neonates following continuous positive airway pressure. Pediatr Dermatol.

[7] Ligi I, Arnaud F, Jouve E, Tardieu S, Sambuc R, Simeoni U (2008). Iatrogenic events in admitted neonates: a prospective cohort study. Lancet..

[8] Kamper J, Ringsted C (1990). Early treatment of idiopathic respiratory distress syndrome using binasal continuous positive airway pressure. Acta paed Scand.

[9] Staudt MD, Etarsky D, Ranger A (2016). Infected cephalohematomas and underlying osteomyelitis: a case-based review. Childs Nerv Syst.

[10] Dahl KM, Barry J, DeBlasi RL (2002). Echerichia hermannii infection of a Cephalohematoma: case report, review of the literature, and description of a novel invasive pathogen. Clin Infect Dis.

